# Experimental Study on High-Performers Quaternary Copolymer Based on Host–Guest Effect

**DOI:** 10.3390/polym13172972

**Published:** 2021-09-01

**Authors:** Tao Xu, Jincheng Mao, Yang Zhang, Xiaojiang Yang, Chong Lin, Anqi Du, Heng Zhang, Quan Zhang, Jinhua Mao

**Affiliations:** State Key Laboratory of Oil and Gas Reservoir Geology and Exploitation, Southwest Petroleum University, Chengdu 610500, China; 15827742134@163.com (T.X.); xjyang@swpu.edu.cn (X.Y.); 201999010016@swpu.edu.cn (C.L.); 13880147377@163.com (A.D.); 201811000155@stu.swpu.edu.cn (H.Z.); zhangsanfengdbsy@163.com (Q.Z.)

**Keywords:** host–guest strategy, hydrophobically associating water-soluble polymers, rheological behaviors, salt tolerance, *β*-cyclodextrin

## Abstract

A quaternary polymer (HGP) was prepared by the free-radical polymerization of acrylamide, acrylic acid, maleic anhydride functionalized *β*-cyclodextrin (MAH-*β*-CD), and N-(3-methacrylamidopropyl)-N, N-dimethylnaphthalen-1-aminium chloride (NAP). It was found that host–guest behavior occurred most effectively at a molar rate of NAP and CD with 1:1, which exhibited better solubility than hydrophobically associative polymer. Moreover, the as-prepared polymer has superior salt tolerance, shear resistance, and viscoelasticity due to host–guest strategy. More importantly, the HGP solution simulates the distribution of formation water in the Bohai SZ1-1 oilfield has good rheological properties at 120 °C. All results show that the proposed polymer could be a competitive candidate in oilfield applications such as fracturing fluids, displacement fluids, and drilling fluids.

## 1. Introduction

Water-soluble polymers have become increasingly important in oilfield applications such as hydraulic fracturing, enhanced oil recovery, and drilling [1]. As the petroleum industry taps deeper reservoirs, there is an urgent requirement that chemicals should maintain performances in harsh reservoirs with high temperatures and salinities [2,3]. Polyacrylamide and partially hydrolyzed polyacrylamide (HPAM) are widely used in polymer flooding and hydraulic fracturing [4]. However, HPAM with poor heat resistance and salt tolerance cannot withstand the harsh reservoir [5,6,7,8].

In addition, scholars have paid increased attention in recent decades to hydrophobically associating water-soluble polymers (HAWSPs), which have good thickening and salt resistance. Hydrophobic association between the hydrophobic groups of HAWSP increases the hydrodynamic volume and increases viscosity significantly [9,10]. Compared with conventional water-soluble polymers, HAWSPs have poor solubility due to the presentence of hydrophobic groups, which one of the factors restricting its application in oil fields.

The host–guest effect has attracted attention in recent years because it can improve the performance of polymers. *β*-cyclodextrin (*β*-CD) is the most used in this field [11,12,13,14]. *β*-CD can form supramolecular structures with guest chains by hydrogen bonding and van der Waals forces [15,16]. There is a network structure in such a polymer solution caused by the inclusion of the *β*-CD and the guest such as phenyls, long-chain alkyls, adamantyl, and azobenzene [17,18,19,20,21]. Weickenmeier et al. obtained a polymer solution with an apparent supramolecular network structure attributed to the host–guest effect between *β*-CD polymers and hydrophobically modified polymers [22]. Wei Bing et al. grafted a *β*-CD and an adamantane ring structure on the side chain of the polymer; the superior properties make this novel polymer promising in enhancing oil recovery [23]. Zou CJ et al. prepared a new supramolecular retarded acid system by *β*-CD and 2-Phosphonobutane-1, 2, 4-Tricarboxylic Acid, which can decrease the corrosion rate with formations [24]. Due to the spatial position of the host–guest molecules on the polymer chain and the difference in their molecular activation energy, the rheological behavior of the polymer solution system is significantly different. According to previous studies, grafting of *β*-CD groups in a HAWSP can produce host–guest interactions, improving properties such as solubility and temperature, salt, and shear resistance [25,26].

**Figure 1:**
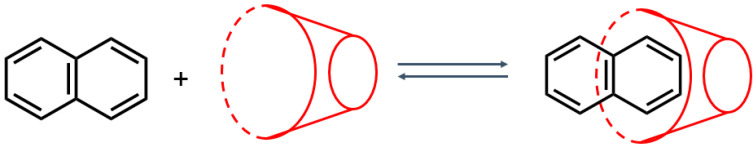
Cyclodextrin and naphthalene have host–guest effects.

Inspired by the above, a quaternary polymer (HGP) was designed by copolymerizing acrylamide (AM), acrylic acid (AA), and two kinds of monomer: *N*-(3-methacrylamidopropyl)-*N,N*-dimethylnaphthalen-1-aminium chloride (NAP) and modified maleic anhydride (MAH-*β*-CD). NAP and MAH-β-CD were selected as functional monomers for the following reasons: (1) the hydrophobic cationic monomer NAP can form hydrophobic microdomains in an aqueous solution due to hydrophobic association, which significantly increases the viscoelasticity. (2) Due to the poor solubility of hydrophobically associating polymers, the introduction of cyclodextrin can speed up the dissolution of the polymer. At the same time, NAP and MAH-β-CD have host–guest effects in the solution. It is reported that the envelope constants between cyclodextrin and naphthalene are 4.67 × 10^3^ molL^−1^ (Figure 1) [27]. (3) In addition, NAP and MAH-*β*-CD are rigid groups, which not only increase the rigidity of the backbone of the polymer but also improve the temperature resistance of the polymer. 

HGP embraces good solubility, thickening performance, shear tolerance, and salt and temperature resistance due to the host–guest effect.

## 2. Experimental Section

### 2.1. Materials

*β*-cyclodextrin(*β*-CD, BR 99%), maleic anhydride (MAH, AR 99%), acetone (AR 98%), chloroform Dimethylamino propyl methacrylamide (DMAPMA, AR 98%), 1-Chloromethyl naphthalene (AR 98%), Anhydrous methanol (AR 99.5%) and acetonitrile (AR 99.5%), Sodium carbonate (AR 99.5%), Ethyl ether, Acrylamide(AM, AR 98%), Acrylic acid (AA, AR 99.5%), 2,2′-azobis (2-methylpropionamidine) dihydrochloride (V50, AR 98%), sodium hydroxide, sodium chloride (NaCl, AR 99%), potassium chloride (KCl, AR 99%), Calcium chloride anhydrous (CaCl_2_, AR 99%), and magnesium chloride (MgCl_2_, AR 99%) were purchased from Chengdu Kelong Chemical Reagents Corp. (Chengdu, China). HPAM (Mw = 16 to 18 × 10^6^) was purchased from the Hengju Oil Field Chemical Reagents Co, Ltd. (Beijing, China). Deionized (DI) water was obtained from a water purification system.

#### 2.1.1. Synthesis of MAH-β-CD

**Scheme 1:**
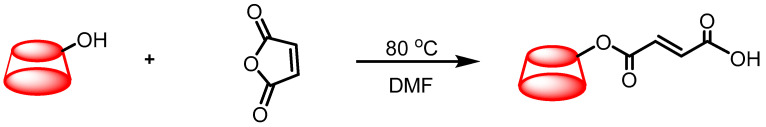
Synthesis route of maleic anhydride cyclodextrin (MAH-*β*-CD).

The activity of various hydroxyl groups in cyclodextrin is different. According to nucleophilic properties, the reaction activity order of the three hydroxyl groups is C6 > C2 > C3. The steric hindrance of the 6-hydroxyl group is small, and the reaction activity is higher under acidic conditions. Therefore, the electrophilic reagent attacks the 6-hydroxyl group, and the reagent with lower reaction activity has the highest selectivity on the 6-primary hydroxyl group. In addition, through references [28,29], it was found that when the molar ratio of 1:1 for the CD and MAH, the yield was higher. A mixture of *β*-CD (5.68 g, 0.005 mol), MAH (4.90 g, 0.005 mol), and anhydrous *N,N*-dimethyl formamide (30 mL) was stirred at 80 °C for 10 h. Then, the reaction mixture was cooled to room temperature, and a large amount of chloroform was added to precipitate the compound. The precipitate was filtered and washed three times with a large amount of acetone. Vacuum drying the precipitate at 75 °C for 24 h resulted in a 62% yield of a yellow powder (MAH-*β*-CD) (Scheme 1).

#### 2.1.2. Synthesis of NAP

**Scheme 2:**
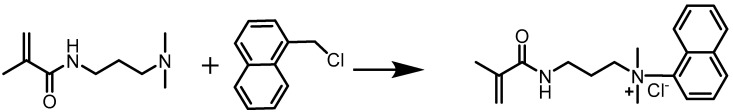
Synthesis route of NAP.

A mixture of (10.215 g, 0.06 mol) DMAPMA and (10.598 g, 0.06 mol) 1-(chloromethyl) naphthalene was added to 60 mL anhydrous methanol and 60 mL acetonitrile, and sodium carbonate (2.11 g) was added. The system was protected by argon and reacted at 60 °C for 48 h at reflux. After the reaction, the solid was filtered, and methanol and acetonitrile were removed at 55 °C by rotary evaporator. The concentrated substance was added to diethyl ether; there white powder was produced, named NAP (yield 91%) (Scheme 2).

#### 2.1.3. Synthesis of Copolymer

**Scheme 3:**
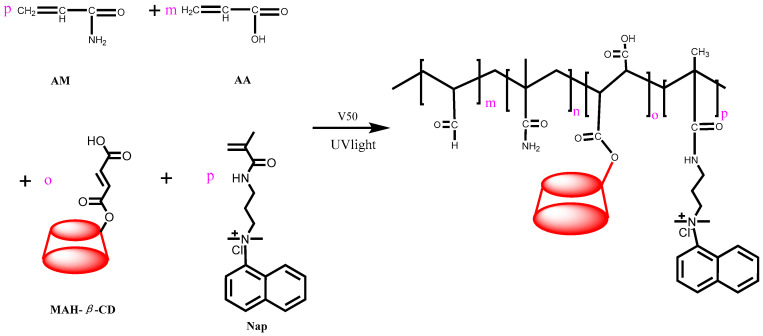
Synthesis route of copolymer.

The appropriate amounts of AM (10 g, 0.14 mol), AA (2.5 g, 0.03 mol), MAH-*β*-CD (0.56 g, 4.54 × 10^−4^ mol), and NAP (0.15 g, 4.54 × 10^−4^ mol) were stirred into deionized water under a nitrogen atmosphere for 30 min, and the total monomer concentration was maintained at 30 wt.%. Then, the pH was adjusted to 7.0–8.0 with sodium hydroxide. After complete dissolution, V50 solution was added, the content of which was 0.035 wt.% of the total mass of the monomer. At last, the solution was under a UV light fixture (T5 8-W UVB) at 25 °C for 8 h (yield 89–92%). The product was purified three times by precipitation with ethanol dried at 25 °C. In this study, three copolymers, HGP, HP-T, and GP-T, were prepared, in which the molar ratio of MAH-*β*-CD to NAP in the three copolymers was 1:1, 1:0, and 0:1, respectively. The synthesis route is shown in Scheme 3.

### 2.2. Characterization

The ^1^H NMR spectra of NAP, HGP, and MAH-*β*-CD were measured on a Bruker AVANCE III HD 400 spectrometer (Bruker BioSpin, Switzerland). The Fourier Transform Infrared Spectroscopy (FT-IR) of HGP was measured with Nicolet 6700 spectrophotometer.

### 2.3. Effect of β-CD and MAH-β-CD on NAP CMC

The influence of *β*-CD and MAH-*β*-CD concentration on the surface tension of NAP was measured by KRUSS DSA30S tensiometer at 25 °C. The measurements were repeated three times independently, and the error was represented by the error strip in the figure.

### 2.4. Effect on the Viscosity of the Functional Monomer

To investigate the effect of the molar ratio of the MAH-*β*-CD and NAP on the viscosity of polymer solutions, the polymer with different molar ratio of MAH-*β*-CD to NAP was prepared. The measurement was repeated three times independently.

### 2.5. Conductivity

In order to explore the impact of the host–guest effect on copolymers’ solubility, the DDS-307^+^ conductivity meter (Chengdu Century Ark Technology Co., Ltd., Chengdu, China) was used to measure the conductivity of HGP, GP-T, and HP-T, and all experiments were conducted at 25 °C. The measurement was repeated three times independently.

### 2.6. Thickening Performance

The viscosities of the copolymer solutions with various concentrations were measured by HAAKE RS600 rheometer (Thermo Fisher Scientific, 81 Wyman Street, Waltham, MA, USA) at 170 s^−1^ and 25 °C. The measurement was repeated three times independently.

### 2.7. Microstructure Analysis

The microstructure of the HGP, HP-T and GP-T solutions was observed by environmental scanning electron microscope (ESEM; Quanta 450, Hillsboro, OR, USA). All samples were frozen at −50 °C using liquid nitrogen, and the frozen surfaces of the samples were observed with the ESEM operating at an accelerating voltage of 20 kV.

### 2.8. Viscoelasticity

The viscoelasticity of copolymers solutions was measured by an Anton Paar rheometer (MCR302) with CP50-1-SN30644 plate fixture (diameter = 0.099 mm) at 25 °C. The measurement was repeated three times independently.

### 2.9. Shear Tolerance

The shear tolerances of the copolymer solutions (0.6 wt.%) were measured by a continuous shear from 7.31 s^−1^ to 1000 s^−1^ at 25 °C for 30 min by the HAAKE MAR III RS 600 rheometer. The shear recoveries of shear rates at 170 s^−1^ and 510 s^−1^ were measured under the same conditions. The measurement was repeated three times independently. The thermal shear of HGP, HP-T, and GP-T solutions was investigated. The temperature was raised from 30 °C to 120 °C over a period of 120 min at a constant shear rate of 170 s^−1^.

### 2.10. Salt Tolerance

To investigate the salt tolerance of the copolymer solutions HGP, HP-T, GP-T, and HPAM, the viscosity of polymers (0.6 wt.%) in different concentrations of sodium chloride was measured. Similarly, the salt tolerance of the copolymers was investigated in calcium chloride solution and magnesium chloride solution, respectively. The viscosity was measured by the HAAKE rheometer at 170 s^−1^ and 25 °C.

## 3. Results and Discussion

### 3.1. Characterization

**Figure 2:**
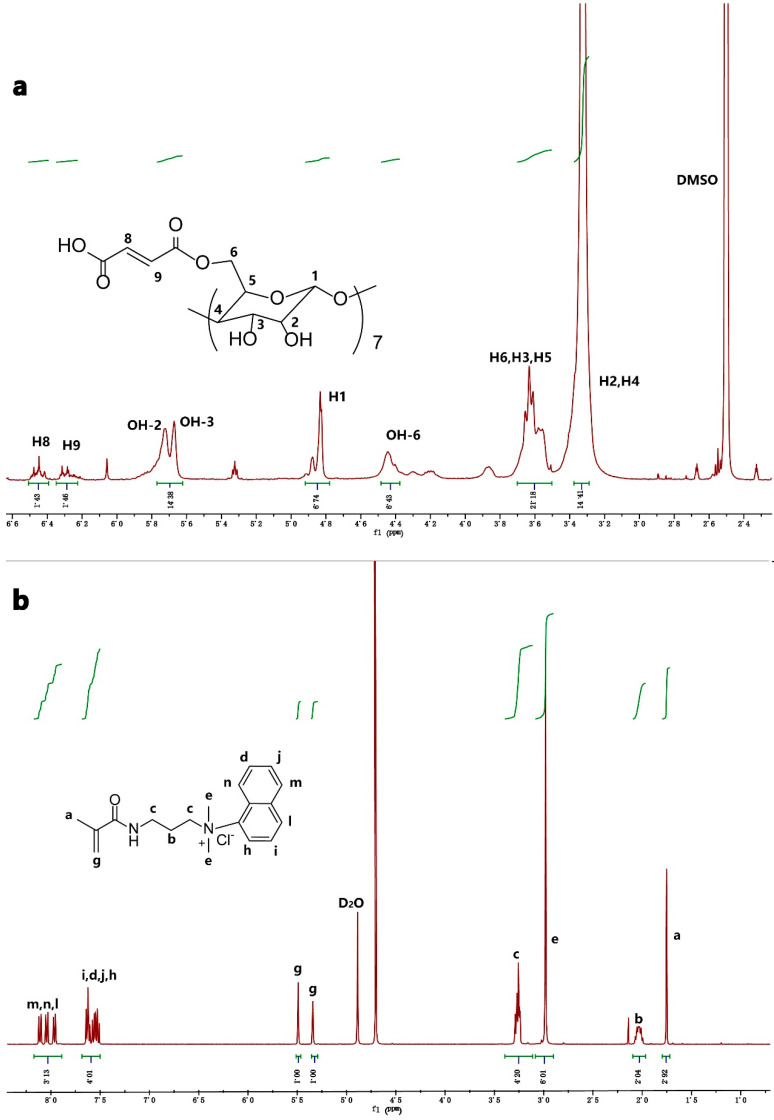

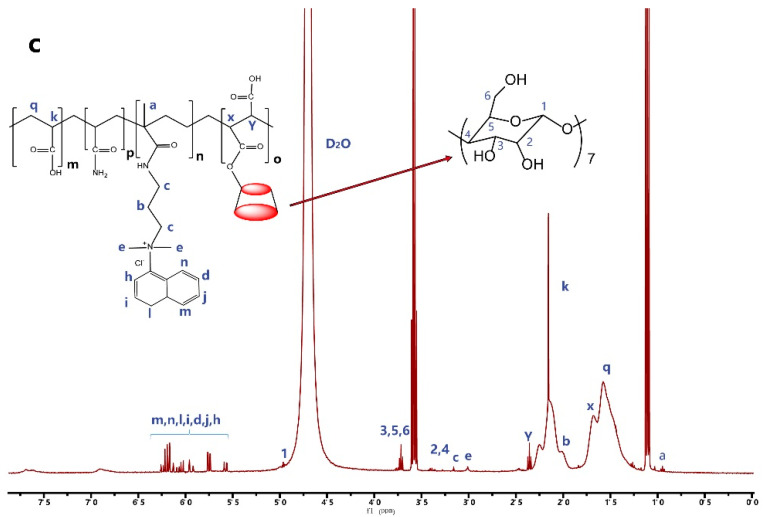
^1^H NMR spectrum: (**a**) MAH-*β*-CD, DMSO; (**b**) NAP, D_2_O; (**c**) HGP, D_2_O.

The HGP structure was confirmed by ^1^H NMR, as shown in Figure 2c. The proton signals at 4.70 ppm were assigned to the solvent proton (D_2_O). Proton signals of 3.0 ppm were assigned to –N^+^–CH_3_, and 5.56 to 6.24 ppm were associated with the proton peak of naphthalene. A 0.88 ppm proton signal could be assigned to the -CH_3_ proton in the polymer backbone. The proton signals of 1.603 ppm and 2.143 ppm were attributed to the -CH_2_-CH- in the polymer backbone. The two protons of the main chain of the MAH-*β*-CD were around 2.45 ppm (-CH-) and at 1.70 ppm (-CH_2_-). The proton signal at 4.998 ppm was associated with the *β*-CD glucose unit (O-CH-O). The mass signal was considered to be the glucose proton units C-CH-OH and C-CH_2_-OH at 3.681 ppm. The 3.272 ppm proton signal was derived from the C-CH-O of the glucose proton unit, which corresponds to Figure 2a,b; the experimental results verified the identity of the synthesized polymer to the target.

**Figure 3:**
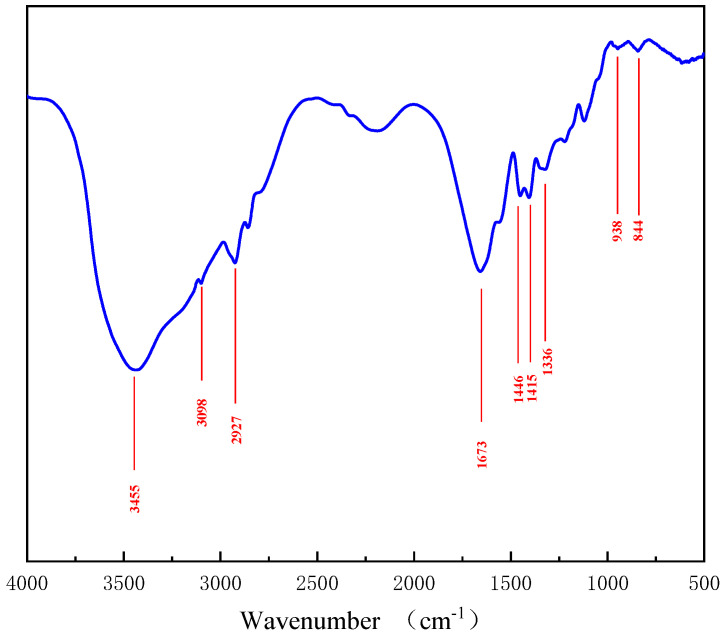
The FTIR spectrum of HGP.

HGP was characterized and confirmed by the FT-IR spectrum shown in Figure 3. The absorption bands at 3455 cm^−1^ and 1673 cm^−1^ were due to the stretching vibration of N-H and C=O in the acrylamide groups (–CONH_2_). The band at 3098 cm^−1^ was attributed to the C-H stretching vibration of arene hydrocarbons. The band at 2927 cm^−1^ was due to the stretching vibration peak of -CH_2_-. The peak at 1446 cm^−1^ was assigned to the stretching vibration of the arene C-C skeleton. The 1415.4 cm^−1^ peak was attributed to the in-plane bending vibration peak of amide C-N and N-H. The bands observed at 1336 cm^−1^ was due to the vibration of -CH_2_-. The band at 844 cm^−1^ was the in-plane and out-plane bending vibration of arene C-H. Furthermore, the stretching vibration of C-O-C referred to the band at 938.00 cm^−1^ which corresponded to the skeleton vibrations of *β*-CD. The FT-IR spectra confirmed that NAP and MAH-*β*-CD had been successfully introduced into the polymer.

### 3.2. Effect of β-CD and MAH-β-CD on NAP CMC

**Figure 4:**
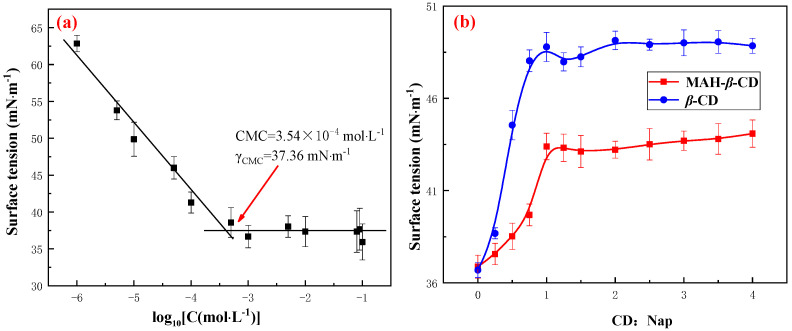
(**a**) Surface tension of NAP at 25 °C; (**b**) relationship between CD:NAP and NAP surface tension at 25 °C.

The critical micelle concentration (CMC) of NAP was measured at 25 °C. As shown in Figure 4a, with the increase in concentration, the surface tension of the NAP solution decreases continuously. When the concentration is 3.54 × 10^−4^ mol/L, the surface tension drops to the minimum and rarely changes with the increase in concentration, indicating that the CMC of NAP is about 3.54 × 10^−4^ mol/L. Figure 4b shows the relationship between the surface tension of NAP (3.54 × 10^−4^ mol/L) and the concentration of cyclodextrin (*β*-CD or MAH-*β*-CD) at 25 °C. Clearly, with the increase in CD/NAP, the system surface tension increases continuously. When CD:NAP ≈ 1, the surface tension reaches the maximum and tends to be stable. It can be seen that MAH-*β*-CD, like the *β*-CD, has a similar binding effect on hydrophobic guest NAP, and an inclusion ratio of CD:NAP ≈ 1.

### 3.3. Effect on the Viscosity of Host–Guest Monomer Ratio

**Figure 5:**
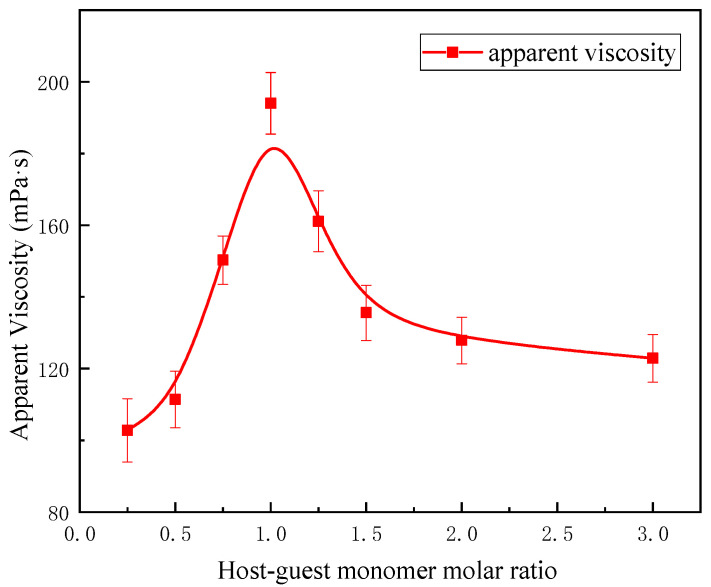
The 0.6 wt.% HGP viscosity at various monomer molar ratios with a shear rate of 170 s^−1^ at 25 °C.

It is well known that monomer content has a marked effect on polymer viscosity [30]. As shown in Figure 5, with the increase in the molar ratio of the host–guest monomer, the viscosity of the solution increased first and then decreased. When the molar ratio of MAH-*β*-CD to NAP was 1:1, the solution viscosity reached the maximum 194 mPa∙s, corresponding to the effect of CD on NAP CMC. The highest effective host–guest structure in the solution occurred when the molar ratio was 1:1, resulting in a significant increase in viscosity. 

### 3.4. Conductivity

**Figure 6:**
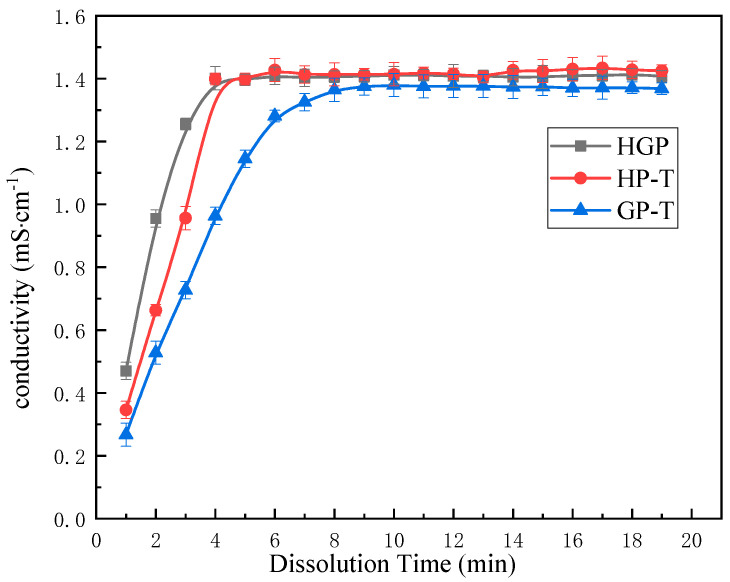
Conductivity changes in the dissolution process of copolymers at 25 °C.

It is important for application in oilfields that polymers dissolve in water immediately. When the polymer is completely dissolved, the conductivity hardly changes over time. As shown in Figure 6, the conductivity of HGP, GP-T, and HP-T gradually stabilized over time. The conductivity of HGP and HP-T tends to stabilize quickly, at 4.2 min and 5.1 min, respectively. However, the GP-T was relatively slow and did not become stable until 8.0 min. *β*-CD can wrap the hydrophobic groups to form an inclusion compound due to its unique structures, including hydrophobic inner cavity and hydrophilic outer cavity. Thus, the hydrophobic association between the hydrophobic groups in the polymer was shielded, accelerating the diffusion rate of the polymer molecules to water, and reducing the dissolution time of the polymer. From this perspective, polymers using the host–guest strategy have more promising application prospects than hydrophobic associating polymers.

### 3.5. Thickening Performance

**Figure 7:**
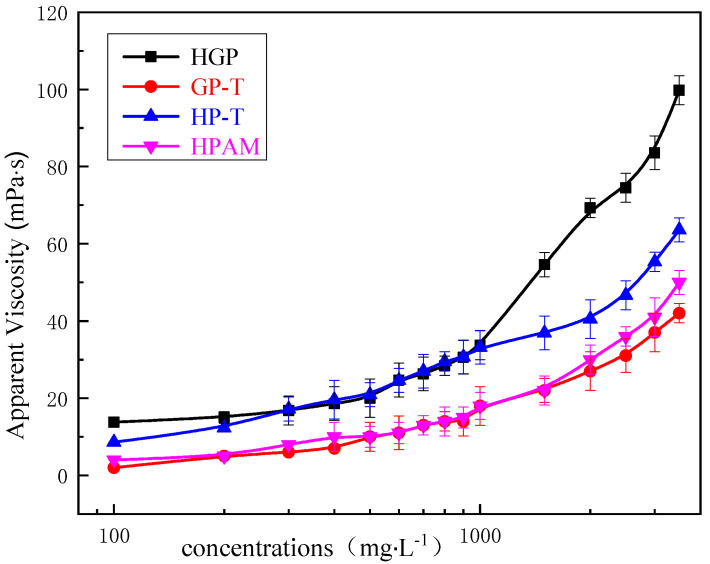
Apparent viscosity as a function of copolymer concentrations for HGP, GP-T, HP-T, and HPAM at shear rate of 170 s^−1^ at 25 °C.

**Figure 8:**
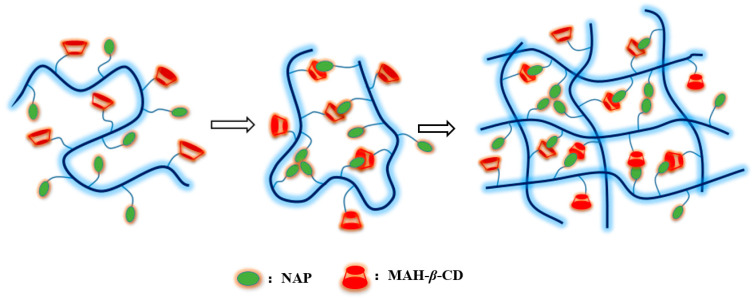
The thickening mechanism of HGP.

The viscosity of the polymer solution increases as the concentration increases. As shown in Figure 7, the four polymers exhibited good proportional linear thickening properties. When the concentration increased from 1000 mg/L to 3500 mg/L, the growth rate in viscosity of the HGP was the highest (168%), and HP-T had the lowest liquid viscosity growth rate (92.5%). This could be interpreted as follows: at low concentrations, the system thickening effect was not obvious, but when the concentration was more than 1000 mg/L, the inclusion effect became significant, and the host–guest recognition between the *β*-CD and NAP on the polymer formed a cross-linked 3D network structure. In addition, the hydrophobic association also contributes to viscosity, as shown in Figure 8. As a result, the viscosity of HGP rose sharply.

### 3.6. Microstructure Analysis

**Figure 9:**
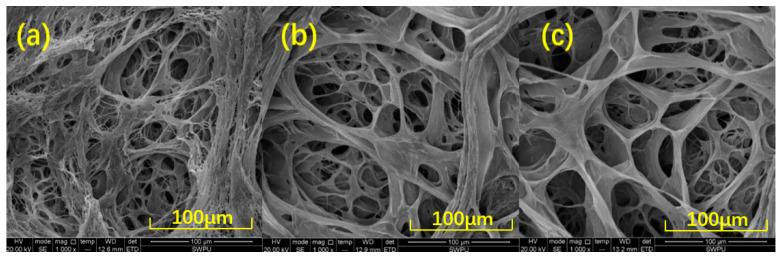
SEM morphologies of 0.6 wt.% GP-T, HP-T, and HGP in DI water (1000×). (**a**) HGP, (**b**) HP-T, (**c**). GP-T.

ESEM was used to investigate the reasons for the increase in polymer viscosity. In Figure 9, microcosmic network structures in HGP are much denser than those of GP-T and HP-T. As shown in Figure 9a, there is a noticeable multilayer 3D structure containing pores with different sizes in the HGP solution as a result of the host–guest effect, which could not only support the polymer chain but also encapsulate a lot of water. In other words, the host–guest effect can increase the apparent viscosity and deformation resistance.

### 3.7. Viscoelasticity

**Figure 10:**
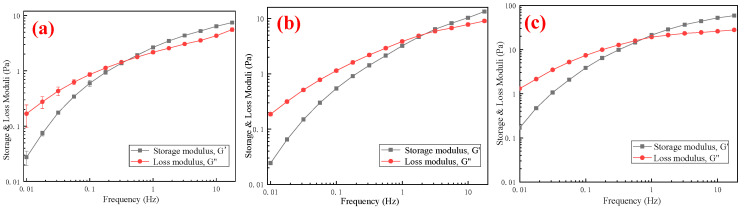
*G’* and *G”* varying as a function of angular frequency for three polymers. (**a**) HGP, (**b**) GP-T, (**c**) HP-T.

The change in storage modulus (*G’*), loss modulus (*G”*), and intersection (*G_c_*) depended on the frequency [4]. Figure 10 shows that for HGP, HP-T, and GP-T, at a low frequency where the *G’* was less than *G”*, the viscous modulus played a dominant role. The storage and loss moduli increased with the increase in frequency. As the scanning frequency further increased above the characteristic frequency where *G’* and *G”* crossed each other (*G_c_*), the *G’* was more significant than the *G”*, indicating that the *G’* played a dominant role. The relaxation time *t_c_* corresponding to the intersection *G_c_* can be used to describe the solution viscoelasticity. A longer *t_c_* verifies that the solution contributes more to elastic efficiency. Table 1 shows the relaxation times of different solutions according to the calculation results; the *t_c_* of HGP, GP-T, and HP-T are about 2.68, 0.451, and 1.3103 s, respectively, suggesting that the host–guest structure can endow the polymer solution with superior viscoelasticity.

### 3.8. Shear Tolerance

**Figure 11:**
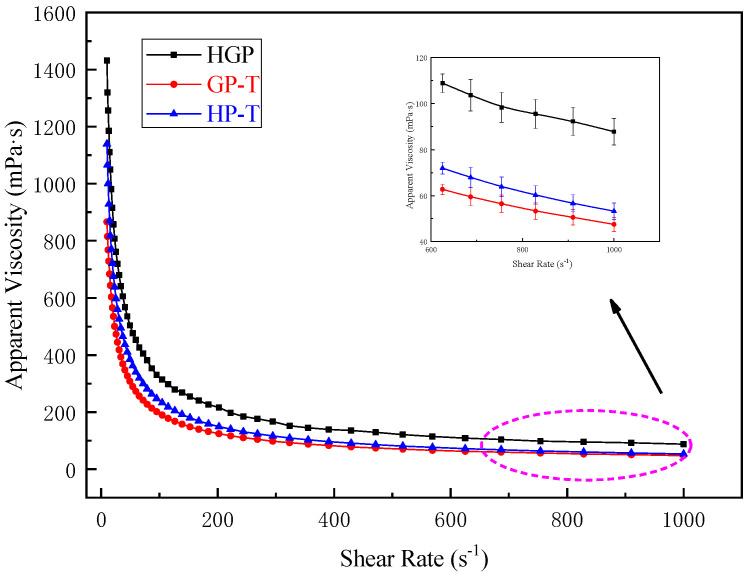
Apparent viscosity relative to shear rate of the HGP, GP-T, and HP-T at 0.6 wt.%.

**Figure 12:**
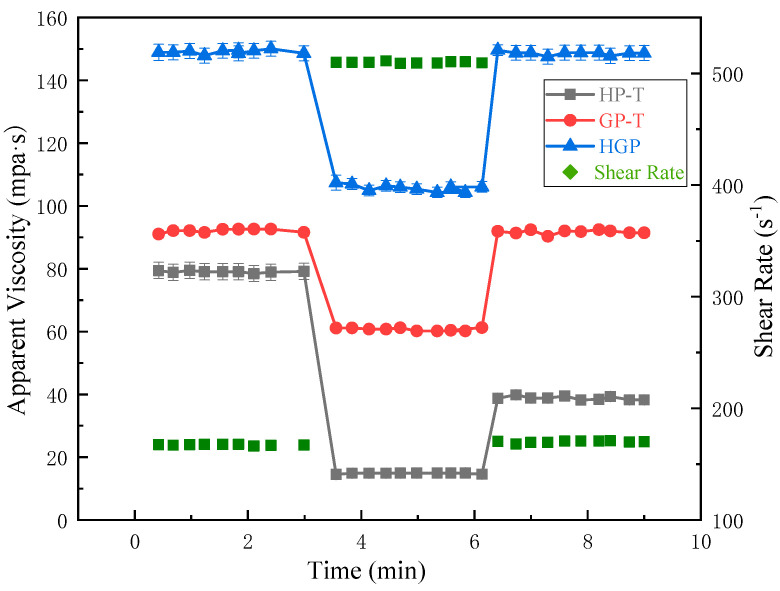
The HGP, GP-T, and HP-T apparent viscosity relative to alternating shear rate.

As shown in Figure 11, the viscosity decreased as the shear rate increased, indicating that those polymer solutions are typical pseudoplastic fluids. With the increase in shear rate, the forces between groups and molecular chains were destroyed, including hydrogen bonds, van der Waals forces, and hydrophobic associations. The flow resistance of the system dropped dramatically, resulting in a decrease in apparent viscosity. In addition, the viscosities of HGP, GP-T, and HP-T were 87.8 mPa·s, 47.5 mPa·s, and 53.2 mPa·s, respectively, at a shear rate of 1000 s^−1^. A similar situation can also be observed in Figure 12. When the shear rate alternated between 170 s^−1^ and 510 s^−1^, the HGP solution showed the best shear recovery performance, indicating that the HGP solution had good shear resistance properties caused by the interaction between the *β*-CD and the hydrophobic group. When the shear occurred, the host–guest interaction was destroyed, producing many free host and guest groups at the cross-section. While shear rate decreased, the host–guest effect reformed, leading to the recovery in the viscosity of polymer solutions.

The polymer will experience shear and temperature increase in the reservoir. Thus, it is necessary to evaluate the behaviors of polymers in formation water. In the experiment, formation water of the SZ1-1 oilfield in the Bohai Sea was used to prepare 0.6 wt.% polymer solution at 25 °C; the formation water composition is shown in Table 2.

**Figure 13:**
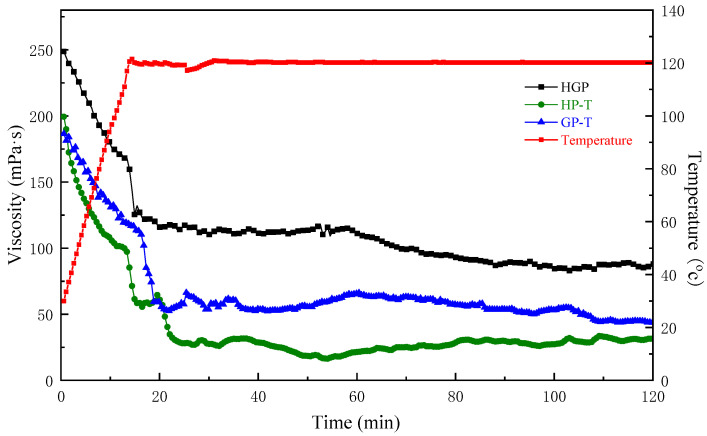
Rheological properties of copolymers HGP, HP-T, and GP-T at 120 °C.

Figure 13 shows that the viscosity of the polymer solution decreased as the temperature increased. From 30 °C to 120 °C, the HGP viscosity maintains at approximately 90 mPa·s, significantly greater than HP-T (51 mPa·s) and GP-T (30 mPa·s), indicating that the HGP has greater temperature resistance than HP-T and GP-T. *β*-CD encapsulating the hydrophobic chain was an exothermic reaction [24,31], leading to the dissociation of the *β*-CD inclusion complex with increasing temperature due to the Brownian motion. In addition, the weakening of the hydrophobic association resulted in a considerable reduction in viscosity. However, the conformation of the polymer at high temperature determines its hydrodynamic volume, which determines the viscosity. The conformation is mainly affected by the rigidity of the polymer and the forces between groups and molecular chains. Although rigidity of the polymer worsens the flexibility of the molecular chain, it can make the molecular chain more extended [32]. Therefore, increasing the rigidity of the polymer can effectively increase the temperature resistance, and the introduction of the hydrophobic group can also change the conformation of the polymer at high temperatures [33]. Thus, the temperature resistance of the polymer solutions at high temperatures was HGP > GP-T > HP-T.

### 3.9. Salt Tolerance

**Figure 14:**
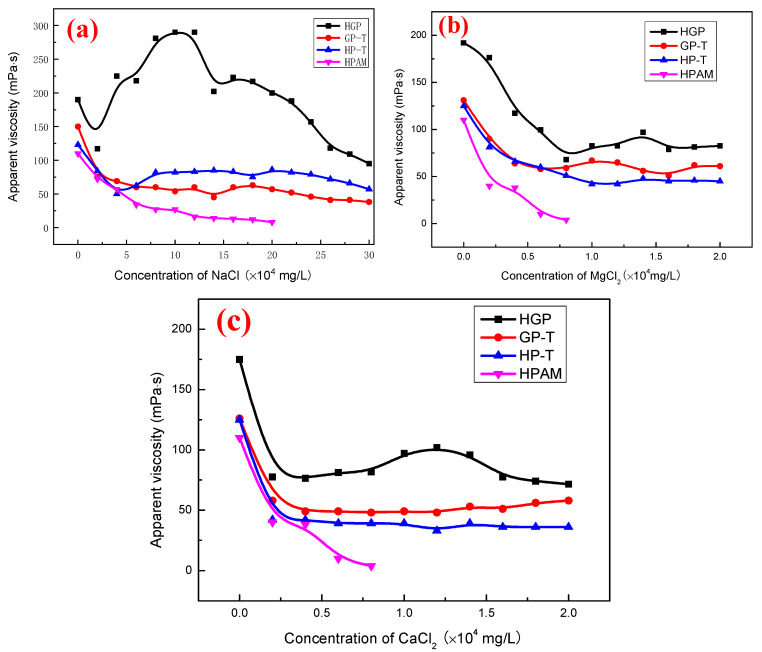
Effects of inorganic salt concentrations on the viscosities of HGP, GP-T, HP-T, and HPAM, 0.6 wt.%. (**a**) NaCl, (**b**) MgCl_2_, (**c**) CaCl_2_.

Inorganic salt has a negative influence on the polymer solution viscosity, especially the divalent ones, such as Ca^2+^ and Mg^2+^ [34]. As shown in Figure 14a, the viscosity change of the HGP solution was more pronounced than those of the HP-T and GP-T solutions. When the NaCl concentration was less than 20,000 mg/L, the viscosity of the HGP solution decreased as the NaCl concentration increased. This can be interpreted as sodium ions shielding the carboxylate ions on the polymer, which reduces electrostatic repulsion between the anions, causing the polymer backbone to curl, resulting in the reduction in viscosity at the initial stage.

When the content of NaCl increases from 2 × 10^4^ mg/L to 12 × 10^4^ mg/L, the polarity of the solution increases, which strengthens the hydrophobic association and the host–guest effect. Macroscopically, the viscosity of HGP increased from 117 mPa·s to 290 mPa·s. Further, when the NaCl concentration reached 30 × 10^4^ mg/L, the viscosity lowered from 290 mPa·s to 89 mPa·s. This can be clarified as when the salt concentration further increases, the electrostatic shielding effect was greater than the host–guest interaction and hydrophobic association, resulting in the polymer backbone crimp and a sharp decrease in viscosity. For HPAM, the electrostatic shielding effect of salt on carboxylate ions was significant, and the polymer backbone crimped seriously, so the viscosity of HPAM is almost the same as that of water [35]. 

Similarly, the effect of CaCl_2_ and MgCl_2_ on the viscosity of the polymer solution was also investigated, as shown in Figure 14b,c. With the increase in concentrations of CaCl_2_ and MgCl_2_, the viscosity of the HGP was higher than that of HP-T and GP-T, indicating that the host–guest effect between NAP and *β*-CD can improve the salt tolerance of the polymer. When the brine concentration reached 2 × 10^4^ mg/L, the viscosity of HGP in CaCl_2_ and MgCl_2_ decreased from the initial viscosity to 71.6 and 82.5, respectively. However, when the concentration of inorganic salts reached 7000 mg/L, the viscosity of the HPAM solution was reduced almost to zero.

## 4. Conclusions

In this study, a water-soluble polymer, HGP, was synthesized that combined the host–guest effect of *β*-cyclodextrin and NAP. From the experiments, six conclusions were drawn:

(1) HGP with good properties was synthesized by free-radical polymerization. NAP, MAH-*β*-CD, and HGP were characterized by ^1^H NMR spectroscopy.
(2) HGP using the host–guest strategy encompasses a better solubility than HAWSPs.
(3) The highest effectiveness of host–guest structures can be formed in an aqueous solution when the molar ratio of *β*-CD to NAP is 1:1.
(4) When the concentration increased from 1000 mg/L to 3500 mg/L, the growth rate in viscosity of the HGP was 168%. The microscopic reason was that microcosmic network structures in HGP are much denser than those of GP-T and HP-T.
(5) The host–guest effect between NAP and MAH-*β*-CD groups improves the performance of HGP, including the shear, viscoelasticity, and salt tolerance.
(6) For the polymer solutions prepared by the formation water of the SZ1-1 oilfield in the Bohai Sea, HGP maintains the viscosity of 90 mPa∙s at 120 °C, 170 s^−1^, which was higher than HP-T and GP-T.

Therefore, HGP has good application prospects for drilling, hydraulic fracturing, and enhanced oil recovery.

## Figures and Tables

**Table 1:** Crossover frequencies and longest relaxation times of three polymer solutions.

	Polymer		
	HGP	GP-T	HP-T
Crossover frequency (Hz)	0.372	2.20	0.767
Relaxation time (s)	2.688	0.451	1.303

**Table 2:** Formation water ion composition.

Composition	Na^+^/K^+^	Ca^2+^	Mg^2+^	CO_3_^2−^	HCO_3_^−^	SO_4_^2−^	Cl^−^
Content (mg∙L^−1^)	3091.64	276.17	158.68	14.21	311.48	85.29	5436.34

## Data Availability

The data presented in this study are available on request from the corresponding author.
